# Edge-Related Loss of Tree Phylogenetic Diversity in the Severely Fragmented Brazilian Atlantic Forest

**DOI:** 10.1371/journal.pone.0012625

**Published:** 2010-09-08

**Authors:** Bráulio A. Santos, Víctor Arroyo-Rodríguez, Claudia E. Moreno, Marcelo Tabarelli

**Affiliations:** 1 Departamento de Botânica, Universidade Federal de Pernambuco, Recife, Pernambuco, Brazil; 2 Centro de Investigaciones en Ecosistemas, Universidad Nacional Autónoma de México, Morelia, Mexico; 3 Centro de Investigaciones Biológicas, Universidad Autónoma del Estado de Hidalgo, Pachuca, Mexico; Centre National de la Recherche Scientifique, France

## Abstract

Deforestation and forest fragmentation are known major causes of nonrandom extinction, but there is no information about their impact on the phylogenetic diversity of the remaining species assemblages. Using a large vegetation dataset from an old hyper-fragmented landscape in the Brazilian Atlantic rainforest we assess whether the local extirpation of tree species and functional impoverishment of tree assemblages reduce the phylogenetic diversity of the remaining tree assemblages. We detected a significant loss of tree phylogenetic diversity in forest edges, but not in core areas of small (<80 ha) forest fragments. This was attributed to a reduction of 11% in the average phylogenetic distance between any two randomly chosen individuals from forest edges; an increase of 17% in the average phylogenetic distance to closest non-conspecific relative for each individual in forest edges; and to the potential manifestation of late edge effects in the core areas of small forest remnants. We found no evidence supporting fragmentation-induced phylogenetic clustering or evenness. This could be explained by the low phylogenetic conservatism of key life-history traits corresponding to vulnerable species. Edge effects must be reduced to effectively protect tree phylogenetic diversity in the severely fragmented Brazilian Atlantic forest.

## Introduction

Understanding how habitat alteration affects biodiversity is a main challenge for ecologists and conservation biologists. Biodiversity has been mostly assessed by simply counting the number of species within an assemblage of organisms; however, this measure assumes that all species contribute equally to the habitat's biodiversity [1]. It is increasingly recognized that biodiversity assessments should include information on the phylogenetic relatedness of species and individuals within assemblages [2]–[4]. This type of information is being used to assign priorities to taxa in conservation evaluations [5]–[7], understand the mechanisms driving patterns of species coexistence and plant community assembly [8], [9], and to determine whether the evolutionary relationships among species of an assemblage are affecting ecological processes, dynamics and ecosystem function [10], [11]. Although the relationship between species extinction and evolutionary diversity is well understood theoretically [e.g. 12], there is little empirical information about the phylogenetic diversity of fragmented tropical forests.

If extinction is a random process, even high rates of extinction may generate little loss in evolutionary diversity [12]. However, empirical evidence from plants, amphibians, birds, and mammals worldwide indicates that extinction and vulnerability to extinction are taxonomically selective [13]–[18]. These nonrandom extinctions have been attributed to evolutionary causes that determine the patterns of rarity across taxonomic groups of different sizes, and to critical aspects of species' life history that constrain their abundance and distribution. These forces are not mutually exclusive given that ecological groups may be phylogenetically clustered, but the ecological causes of nonrandom extinctions are expected to be particularly relevant at smaller spatial scales, especially in species-rich communities with high levels of endemism and species turnover [19]. This is the case for many tropical forests, where the repeated extinction of rare and unique species across landscapes may result in regional and global extinctions. Although an increasing number of papers document drastic reductions in both tree species richness and the diversity of tree life-history traits in fragmented tropical rainforests [20]–[25], to date no studies are evaluating how these changes affect the phylogenetic diversity of the remaining tree assemblages.

Here we use four abundance-based phylogenetic diversity metrics –mean phylogenetic distance (MPD), mean nearest taxon phylogenetic distance (MNTD), net related index (NRI), and nearest taxon index (NTI)− to assess, for the first time, whether the local extirpation of tree species and the functional impoverishment of tree assemblages in fragmented forests may result in a significant loss of tree phylogenetic diversity. MPD measures the average phylogenetic distance among two random individuals drawn from a sample (including conspecifics); MNTD does the same, but the distance is measured to the closest non-conspecific relative [26], [27]. NRI and NTI are standardized metrics of MPD and MNTD, respectively; NRI quantifies the overall clustering of taxa on a tree, while NTI quantifies the extent of terminal clustering, independent of deep level clustering [10], [26], [27].

Given that only a small subset from the original flora is able to persist in fragmented forests, and that the remaining assemblages become increasingly dominated by a few pioneer tree species [21], [23], [28], it is expected that the probability of sampling two conspecific individuals increases after fragmentation, resulting in lower MPD in fragmented forests when compared to continuous ones. MNTD is expected to increase in fragmented forests due to a reduction in species richness: as more species are excluded from the local assemblage, most remaining species will be distant relative of at least one of the already-sampled species [see 26]. The magnitude of such increase, however, will depend on how distant the non-conspecific remaining individuals are in phylogenetic terms. The effect of forest destruction on NRI and NTI will depend on the level of phylogenetic clustering of the original assemblage as well as on the phylogenetic patterns of local species loss. For instance, if the phylogenetic structure of the original assemblage is even and local extinction is phylogenetically overdispersed, then NRI and NTI are likely to remain unchanged.

We tested these predictions using a large vegetation dataset from an old (>200-yr-old) severely fragmented landscape in the Brazilian Atlantic rainforest, where recent studies have demonstrated striking edge-related shifts in tree assemblage composition, structure, and function [22], [23], [25], [29], including a drastic reduction in tree species richness and stem density along forest edges and small forest remnants. We first compared MPD, MNTD, NRI, and NTI across forest edges, small (<80 ha) forest fragments, and old-growth interior areas. Then, we examined how these metrics of phylogenetic diversity varied along a 5 to 65-yr-old chronosequence of forest regeneration as well as the degree of similarity between forest edges, small forest fragments and early- to mid-secondary stands in terms of phylogenetic diversity (a test of the forest degeneration hypothesis sensu Tabarelli et al. [30]). Finally, we used three vulnerable functional groups formed by shade-tolerant, emergent, and large-seeded species to evaluate if the functional impoverishment previously documented for the study area has been paralleled by a loss of tree phylogenetic diversity.

## Methods

The Brazilian Atlantic rainforest represents one of the most important biodiversity hotspots in the world [31]. Originally, it covered around 150 million ha, but recent estimations indicate that less than 16% of the forest remains [32]. In addition to be poorly protected (nature reserves only account for 1% of the original forest), the remaining forest cover is distributed in *ca*. 250000 forest fragments, 80% of which are smaller than 50 ha and the average distance between fragments is *ca*. 1500 m [32]. Furthermore, almost half of the remaining vegetation is less than 100 m from the nearest edge [32].

**Figure 1:**
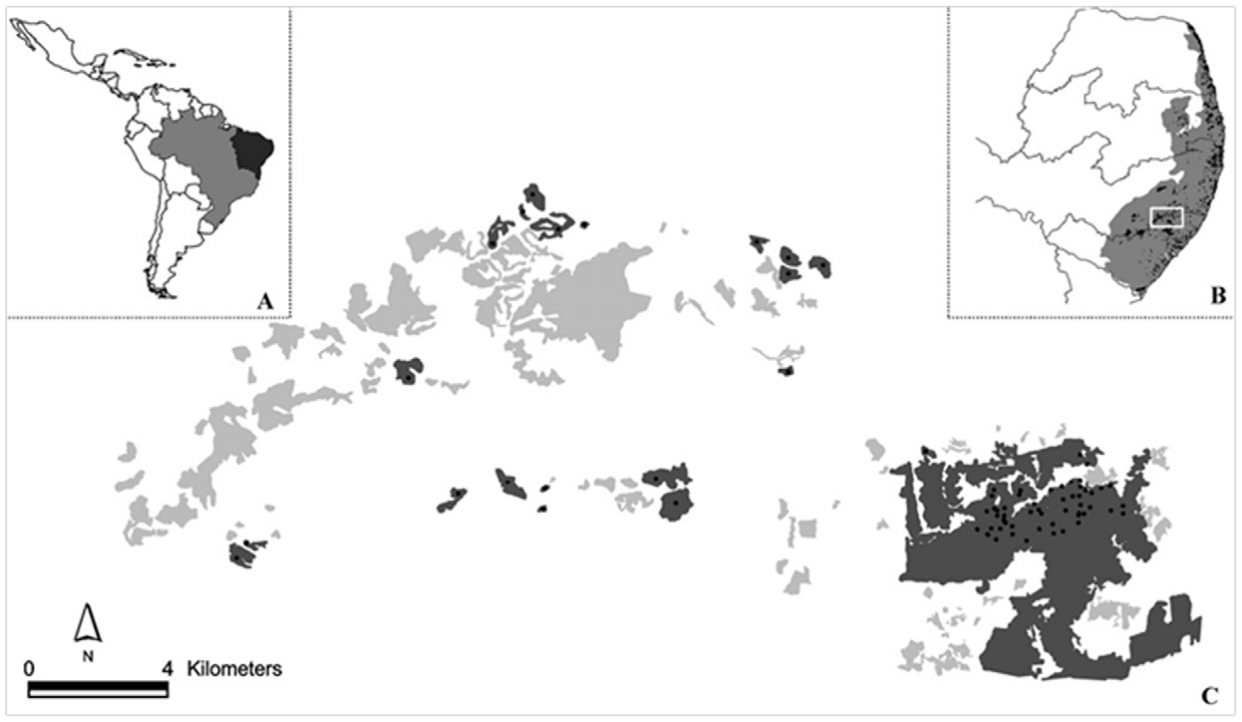
Study landscape at the Brazilian Atlantic forest. (A) Northeastern Brazil, where this study was conducted. (B) Distribution of the Atlantic forest of northeast Brazil ( =  Pernambuco Center of Endemism), note original (grey) and current (black) distribution of this forest in the region; white rectangle represents the study landscape (amplified in C). (C) Study landscape showing the location of 75 plots of 0.1 ha sampled to describe the phylogenetic diversity of tree assemblages in forest edges, small forest fragments, secondary forest patches and old-growth interior forests. Dark shaded polygons represent the forest fragments sampled; lightly shaded and white areas represent the remaining Atlantic forest remnants that were not sampled and a uniform matrix of sugarcane monoculture, respectively.

The study was carried out at the Usina Serra Grande, owned by a large, private sugar company of the same name located in the state of Alagoas, northeastern Brazil (8°30′S, 35°50′W; Figure 1). Information on the climate, soil, fauna and flora of this region is detailed in Santos et al. [23]. This landholding still retains *ca*. 9000 ha (9.2%) of the forest cover assigned to a unique biogeographic region of the Atlantic forest: the Pernambuco Center of Endemism [33]. We selected a large (667 km^2^), severely fragmented landscape containing 109 forest fragments (ranging from 1.7 to 3500 ha), all of which are entirely surrounded by a uniform, stable and inhospitable matrix of sugarcane monoculture. Sugarcane cultivation at Serra Grande dates back to the 19th century, and provides a rare opportunity for Atlantic forest fragmentation studies. The 3500 ha Coimbra forest represents the largest and best preserved forest fragment in the region, and the undisturbed areas in its interior can be used as control sites because they still retain many plant and vertebrate groups typical of vast undisturbed tracts of Atlantic forest [23].

Elsewhere, we have presented detailed descriptions of the methods used to sample the vegetation at Serra Grande [23], hence only a brief overview is given here. All trees ≥10 cm DBH were sampled and identified to the species level in 75 plots with an area of 0.1 ha (10×100 m). Plant vouchers are available at the Federal University of Pernambuco, UFP Herbarium, Brazil (voucher Nos. 34445–36120). The plots were randomly located in four habitats: (i) 20 plots in old-growth forest interior areas of the Coimbra forest at least 200 m from the edge, with no detectable edge influence (control plots); (ii) 10 plots at forest edges along the 39.9-km-long perimeter of the Coimbra forest, starting at the forest edge and penetrating perpendicularly 100 m into the fragment (edge plots); (iii) 20 plots located at the geometric center of 20 small forest fragments (3.4–79.6 ha; fragment plots); and (iv) 25 secondary forest plots, i.e. <2 ha patches of 5 to 65-yr-old secondary-growth forests created by the abandonment of slash-and-burn plots following 5–10 yrs of subsistence agriculture within the Coimbra forest (one plot per forest stand).

Our definitions of forest edges and forest interior areas are based on Laurance et al. [34], who showed that most edge effects penetrate less than 200 m into Amazonian forest fragments. The distance between tree plots and the nearest forest edge was 200–758 m (mean  = 394 m) for control plots, 0 m for edge plots, 72–248 m (mean  = 154 m) for fragment plots, and 32–578 m (mean  = 364 m) for secondary-growth forest plots. Although the edge, secondary, and control plots were all embedded in the Coimbra forest, among-plot variation in tree assemblage composition cannot be attributed to the spatial arrangement of the plots [23]. Also, fragment metrics such as area, shape, and isolation have been shown to be poor predictors of tree assemblage structure and function in that region [23]. We excluded these covariables from our statistical analyses after verifying that none of those metrics correlated with phylogenetic diversity measures.

### Local extirpation of tree species and phylogenetic diversity metrics

The loss of tree species has been well documented in the study area [23], showing that local species richness is significantly lower in forest edges (18.4±4.5 species; mean ± SD per 0.1-ha plot) and small forest fragments (23.7±9.6) than in old-growth forest interior areas (36.8±7.3). Stem density (dbh ≥10 cm) also decreased from 101.6 (±21.7) stems in old-growth interior areas to 73.8 (±25.4) in forest fragments and 59.8 (±7.5) in forest edges. To evaluate the extent to which the local extirpation of tree species from edges and forest fragments has affected the phylogenetic diversity of the remaining tree assemblages we first produced a full species list based on APG III [35] classification after identifying the 5257 trees sampled in the 75 plots. We then classified species by genus and family –we recorded 206 species belonging to 125 genera and 48 families (Table S1)− and used the phylomatic function of Phylocom 4.1 [27] to assemble the species list into a phylogeny. For this, we used the dated tree from Davies et al. [36] available in Phylomatic website, whose branch lengths from the terminals (family names) represent maximum ages for those clades. After constructing the time-calibrated phylogeny of our study area, we used the comstruct function of Phylocom 4.1 to calculate the phylogenetic diversity metrics for each sample. The switch ‘-a’ was used to weight phylogenetic distances by taxa abundances.

To determine whether the phylogenetic structure of forest edges, small forest fragments, and old-growth forest interior areas differed from the phylogenetic community structure expected by chance, we compared observed phylogenetic distances among individuals (MPD and MNTD) to the expected phylogenetic distances for 999 randomly generated null communities (MPD.rnd and MNTD.rnd, respectively). We used null model 2 of Phylocom 4.1 to generate null communities. In this model, species in each sample become random draws from the phylogeny pool [27]; it assumes that all species of the pool are equally able to colonize any habitat within the study area, whether in fragmented or continuous forests.

After computing observed and expected MPD and MNTD for each sample, we calculated NRI and NTI metrics. NRI is defined as [-1 (MPD – MPD.rnd)/MPD.sd)] and NTI as [-1 (MNTD – MNTD.rnd)/MNTD.sd)]; where MPD.sd and MNTD.sd represent the standard deviation of MPD.rnd and MNTD.rnd, respectively, from the 999 null communities [10], [27]. Positive values of NRI and NTI indicate phylogenetic clustering, while negative values represent phylogenetic overdispersion. If the simple null model used to derive these metrics is appropriate, the significance of a pattern is contained in the value of the metrics themselves (< −1.96 is significantly even and >1.96 is significantly clustered) [26]. To corroborate this assumption, we compared NRI and NTI values to the *P*-value estimated for each sample. We divided the number of runs in which the expected mean was smaller or greater than the observed mean by the total numbers of runs (999+1) to calculate the *P*-value [27]. Only NRI and NTI values < −1.96 and >1.96 were associated with *P*-value <0.05, confirming that the criterion mentioned by Vamosi et al. [26] was also adequate to our dataset. We reported MPD and MNTD in millions of years and NRI and NTI in units of standard deviation.

To evaluate if the functional impoverishment previously documented for the study area [22], [23], [25] has been paralleled by a loss of tree phylogenetic diversity, we selected three functional groups that are typically vulnerable to forest fragmentation throughout the Neotropics: shade-tolerant, emergent, and large-seeded vertebrate-dispersed tree species [20], [21], [24], and calculated the proportion of species within each functional group for each plot.

### Statistical analyses

We used one-way analyses of variance (ANOVAs) to test for differences in MPD, MNTD, NRI and NTI among edge, fragment and control plots after checking data normality with the Shapiro-Wilk test. Tukey-Kramer HSD (honestly significance difference) tests were used *a posteriori* to compare habitat means. Non-linear regressions (exponential rise to maximum and exponential decay curves) were used to fit phylogenetic metrics to the age of secondary forests. We used Pearson product-moment correlations to analyze the relationship between phylogenetic diversity metrics and the proportion of shade-tolerant, emergent, and large-seeded vertebrate-dispersed tree species. All statistical analyses were performed using JMP 7.0 (SAS Institute Inc.) and SigmaPlot 10 (Systat Software Inc.).

## Results

Mean phylogenetic distance (MPD) differed significantly among habitats (Table 1). It was similar between fragment and control plots (Tukey-Kramer HSD, *P*>0.05), but was on average 11% lower in edge than in control plots (*P*<0.05; Table 1). Such a reduction represented a pairwise phylogenetic distance of about 19 million years between two randomly chosen individuals in forest edges (Table 1). Mean nearest taxon phylogenetic distance (MNTD) also differed among habitats (Table 1). As MPD, MNTD was similar between fragment and control plots (*P*>0.05), but was on average 17% greater in edge than in control plots (*P*<0.05; Table 1). This percentage represented a 17 million years increase in the phylogenetic distance between a randomly chosen individual and its closest non-conspecific relative in forest edges (Table 1). All fragment, edge and control plots showed net relatedness index (NRI) and nearest taxon index (NTI) between −1.96 and 1.96, indicating that the phylogenetic clustering (or evenness) of the tree assemblages studied did not differ significantly from that of randomly generated null communities. Also, NRI and NTI varied irrespective of habitat type (Table 1).

**Table 1:** Phylogenetic diversity metrics (mean ± SE) of tree assemblages at Serra Grande, northeastern Brazil.

Phylogenetic diversity metric	Habitat type			ANOVA	
	F	E	C	*F*-value	*P*-value
Mean phylogenetic distance (MPD)	187.1±2.9^ab^	174.6±8.1^a^	195.3±3.6^b^	4.867	<0.05
Mean nearest taxon phylogenetic distance (MNTD)	116.2±3.7^ab^	124.5±6.5^b^	106.6±2.7^a^	5.564	<0.05
Net relatedness index (NRI)	0.51±0.13	0.30±0.08	0.19±0.18	1.113	0.337
Nearest taxon index (NTI)	0.33±0.16	0.26±0.24	0.01±0.18	0.894	0.416

F, E, and C represent small forest fragments (*n* = 20), forest edges (*n* = 10), and old-growth forest interior areas (*n* = 20), respectively. MPD and MNTD are expressed in million years; NRI and NTI are in units of standard deviation. Significant differences in post hoc comparisons (Tukey-Kramer HSD tests) between habitat types are indicated by different letters in a same row.

**Figure 2:**
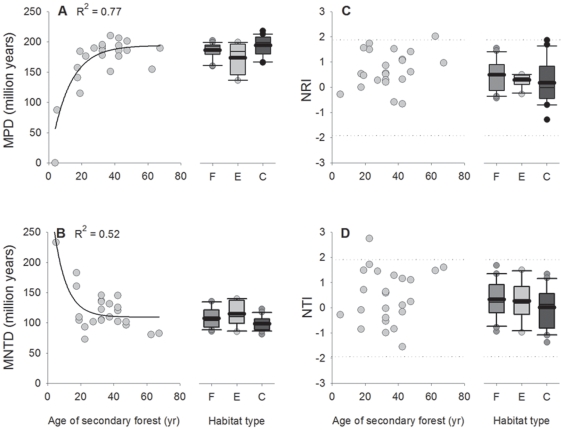
The relationship between forest age and phylogenetic diversity metrics. The relationship between the age of 25 secondary forest patches within the Coimbra Forest and (A) the mean phylogenetic distance (MPD), (B) mean nearest taxon phylogenetic distance (MNTD), (C) net relatedness index (NRI), and (D) nearest taxon index (NTI) at Serra Grande, northeastern Brazil. R^2^ values are shown for significant relationships. The mean (solid line), median (thin line), 25th and 75th percentiles (boundaries of boxes), 10th and 90th percentiles (whiskers above and below box plots), and each outlier (points outside 10th and 90th) are also indicated for equal-sized plots within small forest fragments (F, *n* = 20), forest edges (E, *n* = 10) and old-growth forest interior areas (C, *n* = 20). Values outside the area delimited by dotted lines in plots C and D indicate significant phylogenetic clustering (>1.96) and overdispersion (< −1.96).

MPD increased exponentially with the age of secondary-growth stands and tended to stabilize after 30–40 years of forest regeneration. All MPD values calculated for fragment and edge plots fell inside the overall range of the 5 to 65-yr-old chronosequence (0 to 210.2 million years), but four control plots (20%) had MPD values slightly over 210.2 million years (up to 218.3) (Figure 2A). On average, the MPD of edge and fragment plots was similar to that predicted for a 27-yr-old and 39-yr-old secondary-growth forest, respectively (174.6 and 187.1); control plots had average MPD predicted for >65-yr-old forest stands (195.3) (Figure 2A). MNTD decreased exponentially with the age of secondary-growth stands, but the relationship between forest age and MNTD tended to disappear after 20 years of regeneration. All values calculated for fragment, edge, and control plots fell inside the range of the chronosequence (72.8 to 233.1) (Figure 2B). On average, edge and fragment plots had a MNTD value similar to that predicted for a 21-yr-old and 27-yr-old, respectively (124.5 and 116.2); control plots showed average MNTD similar to that predicted for >65-yr-old forest stands (106.6) (Figure 2B). NRI and NTI did not correlate with the age of second-growth stands (Figure 2C, D) and 23 of the 25 secondary forest plots had NRI and NTI between −1.96 and 1.96 (a 62-yr-old forest stand presented a NRI of 2.01 and a 22-yr-old forest stand showed a NTI of 2.74).

**Figure 3:**
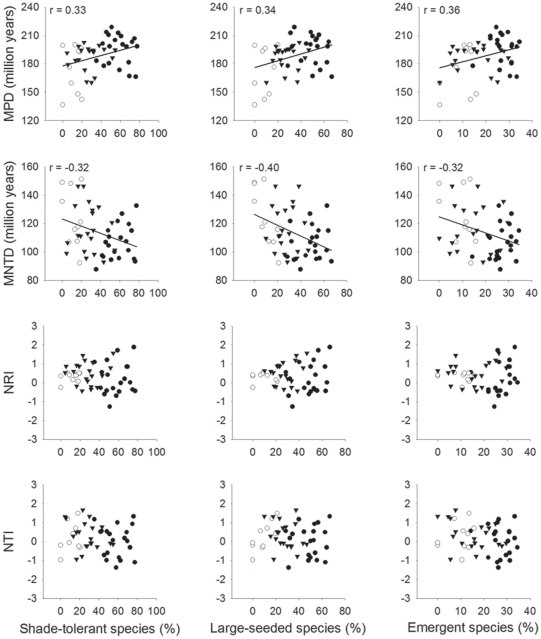
The relationship between functional attributes of tree assemblages and phylogenetic diversity metrics. The correlation between the proportion of species within vulnerable functional groups and the mean phylogenetic distance (MPD), mean nearest taxon phylogenetic distance (MNTD), net relatedness index (NRI), and nearest taxon index (NTI) at Serra Grande, northeastern Brazil. Triangles, open circles, and dark shaded circles represent plots in small forest fragments (*n* = 20), forest edges (*n* = 10), and old-growth forest interior areas (*n* = 20), respectively. Pearson product-moment correlation coefficients are shown for significant relationships (*P*<0.05).

MPD correlated positively and MNTD negatively with the proportion of shade-tolerant, emergent, and large-seeded species, but correlations were weak (r<0.40; see high data dispersion in Figure 3). There was no relationship between the proportion of species within each functional group and NTI or NRI (Figure 3).

## Discussion

In the last two decades many studies have documented the local extirpation of plant and animal species from fragmented tropical rainforests [20], [24], [37], particularly from forest edges [34], [38]. This spatially nonrandom pattern of species impoverishment has pervasive effects on the subsequent community dynamics and ecosystem function [21], [34], but its impact on tree evolutionary diversity has never been examined despite the implications to conservation [5], [10]. One of the major conclusions of this study is that the local extirpation of tree species from forest edges results in a significant loss of tree phylogenetic diversity. Such a loss is observed at the plot scale as a decrease by 11% in the phylogenetic distance between any two randomly chosen individuals and an increase by 17% in the distance between a given individual and its closest non-conspecific relative, indicating that edge effects in the study area are much more profound than previously envisioned and documented. Given that almost half of the remaining hyper-fragmented Brazilian Atlantic forest is less than 100 m from the nearest edge [32], it is likely that the edge-related loss of tree phylogenetic diversity is even more relevant at the regional scale.

The forest edges and forest fragments we studied have been embedded in a stable landscape for as long as 200 years. The fact that forest fragments showed intermediate MPD and MNTD between degraded forest edges and conserved old-growth interior areas provides new insights into the manifestation of late edge effects in fragmented tropical rainforests; a phenomenon that is currently poorly understood owing to the scarcity of long-term data or studies in old fragmented landscapes. On one hand, our results reinforce the notion that edge effects are a continual phenomenon in the Brazilian Atlantic forest that drives small forest fragments toward early- to mid-successional systems [30]. On the other hand, the intermediate condition faced by small forest fragments suggests that even two centuries of fragmentation may not be long enough to allow the full spectrum of edge effects to be seen in their core areas, which already exhibit evidence of many types of edge effects [22], [23], [25], [29]. In fact, the role of greater time lags in the manifestation of fragmentation effects on tree assemblages has received little attention in the habitat fragmentation literature [39]. This oversight arises not only from the misinterpretation of habitat fragmentation as a static phenomenon rather than a dynamic process [40], but also from not considering the exceptionally long lifespan of many old-growth tropical trees (several centuries in some cases [41]).

Another important conclusion of this study is that 200 years of deforestation and forest fragmentation in our study area have not resulted in phylogenetic clustering or evenness of the remaining tree assemblages. There is ample evidence from different Neotropical rainforests that the tree species that disappear first from fragmented landscapes share similar life-history traits [e.g. 20], [ 21], [ 24]. If tree life-history traits have evolved within particular lineages (trait conservatism sensu Cavender-Bares et al. [42]), the local extirpation of tree species in fragmented tropical rainforests will ultimately change the evenness properties of the remaining phylogenetic tree. Our findings show that at least for the trees of Serra Grande this is not the case, as NRI and NTI varied regardless habitat type and both altered and non-altered assemblages showed a random phylogenetic structure based on the regional phylogeny pool (NTI and NRI between −1.96 and 1.96). In fact, local extirpation in this region is likely to have occurred randomly or uniformly (but not in a clustered manner) throughout the phylogenetic tree, following the distribution of key life-history traits [22], [25]. The lack of strong correlations between the phylogenetic diversity metrics and the proportion of species within each functional group also suggests low phylogenetic trait conservatism in the tree assemblages examined, but further studies are needed to properly address this issue.

Given the lack of large forest remnants and the current status of conservation of the Brazilian Atlantic forest, the protection of small forest fragments should be done rather than discussed [24]. That said, we would like to stress that conservation efforts in this biologically unique region and probably other tropical rainforests with similar spatial configuration should focus on mitigating current edge effects and preventing the manifestation of late edge effects in the core areas of their small forest fragments. To that end, it is essential to create buffer zones around the forest remnants and connect them with wide forest corridors to reduce edge effects. Otherwise, the long-term conservation of biodiversity and ecosystem function is at risk.

## Supporting Information

Table S1 Full tree species list of Serra Grande, northeastern Brazil. Total species abundance is shown for small forest fragments (F), forest edges (E), old-growth forest interior areas (C), and secondary forest patches (S).
(0.05 MB XLS)
